# Evaluation of Skin Irritation of Acids Commonly Used in Cleaners in 3D-Reconstructed Human Epidermis Model, KeraSkin^TM^

**DOI:** 10.3390/toxics10100558

**Published:** 2022-09-24

**Authors:** Jee-hyun Hwang, Seungmi Lee, Ho Geon Lee, Dalwoong Choi, Kyung-Min Lim

**Affiliations:** 1 College of Pharmacy, Ewha Womans University, Seoul 03760, Korea; 2 Transdisciplinary Major in Learning Health Systems, Department of Health and Safety Convergence Science, Korea University, Seoul 02481, Korea

**Keywords:** skin irritation, reconstructed human epidermis model, acids, alkalis, pH, cleaner

## Abstract

Cleaners such as dishwashing liquids contain various chemicals that cause skin damage. Alkaline agents used in cleaners alter the lipid composition of the skin and damage the skin barrier. However, little is known about the effects of acids used in cleaners on the skin. Here, we investigated the effects of acidic pH on the skin and evaluated the skin irritation of acids commonly used in cleaners with a 3D-reconstructed human epidermis model, KeraSkin^TM^, according to OECD TG439. First, to examine the effects of acidic pH, we evaluated the skin irritation of citrate buffers (0.1 M, McIlvaine buffer) prepared in a wide pH range (pH 1.5–6.0). Surprisingly, cell viability was not significantly affected even at pH 1.5, reflecting that the acidity alone may not be sufficient to induce skin irritation. Even after longer exposure (180 min), the cell viability was not reduced below 50%, a cutoff to determine an irritant. To examine the effect of the anionic part, several organic acids used in cleaners (citric acid, glycolic acid, lactic acid, malic acid, and succinic acid) were examined. These organic acids also failed to reduce viability at 0.1 M. However, at 1 M, most of the acids tested, except lactic acid, were determined to be skin irritants. Histology further supported the skin irritancy of acids at 1 M. Similarly, inorganic acids (hydrogen bromide, hydrogen chloride, nitric acid, and sulfuric acid) were determined to be irritants only at 1 M. In the case of alkaline agents, pH and concentrations were also important factors to determine the skin irritancy, although the epidermal structure and lipids were more damaged than acids. Collectively, we demonstrated that both the pH and concentration are important factors for the skin irritancy of acids, shedding an important insight into the mechanism of skin irritation.

## 1. Introduction

Cleaners are widely used household chemicals for the purpose of cleaning the surface of utensils, foods, and houses. Cleaning products contain detergents, solvents, acids, bases, and disinfectants, which facilitate the removal of dirt and stains from surfaces, and sanitizes it. Cleaners can be also categorized into acidic and alkaline cleaners depending on the pH [1]. Acidic cleaners, which contain various organic and inorganic acids are useful for removing mineral deposits or oxidation products on surfaces by dissolving alkaline minerals and salts. On the other hand, alkaline cleaners can effectively remove organic stains such as oils, fats, greases, and proteins from the surface [2].

Cleaners can damage the skin by destroying the epidermal lipid barriers. The type, concentration, and combination of detergents, their persistence on the skin, and pH can decide the adverse effects of products with respect to skin dryness and irritation [3,4]. In particular, the strong bases essential for removing animal-derived dirt result in highly alkaline cleaner products, which may be strongly irritating to the skin since the skin itself has a slightly acidic pH, between 4.0 and 6.0 [5,6,7], and the acid mantle of SC is pivotal for the formation of skin barrier function [8]. Indeed, it has been reported that the skin proteins swell markedly if exposed to highly alkaline substances (pH > 8.0). Optical coherence tomography (OCT) images of the stratum corneum (SC) [9] after its exposure to acidic (4.0), neutral (6.5), and alkaline pH (10.0) conditions, showed that there is significantly greater SC swelling when exposed to alkaline pH solutions, while acidic pH induces SC shrinking [10]. Alkaline pH also has an effect on SC lipids, with the ionization of fatty acids in the lipid bilayers causing the overall destabilization of the lipid bilayers [11].

As with alkaline agents, acids are expected to induce skin irritation. Extreme pH (<2.0 or >11.0) is generally considered to be corrosive or strongly irritating to the skin [12]. The United Nations Globally Harmonized System of Classification and Labeling of Chemicals (GHS) recommends the exemption of skin irritation tests for substances within those extreme pH ranges [4,12]. Interestingly, while the skin irritancy of alkaline agents has been well established through many experimental studies [13], the skin irritancy of acids was not clearly characterized with respect to pH, concentration, or types of acids. This is an important point for the manufacture of cleaners, since companies produce cleaners with acidic to neutral pH, in a range of 5.5–7.0, which may have less impact on the structural SC integrity. 

Here, we studied the effect of pH, concentrations, and types of acids on skin irritation using a 3D-reconstructed human epidermis model, KeraSkin™, in accordance with the Organisation for Economic Co-operation and Development (OECD) Test No. 439: In Vitro Skin Irritation: Reconstructed Human Epidermis Test Method. In addition, alkaline agents used in cleaners were prepared at high and low concentrations, and their skin irritancies and effects on the epidermal lipids were compared with those of acids.

## 2. Materials and Methods

### 2.1. Materials

Specific details about the acid and alkali chemicals, with pH 0.1 and 1.0 M, and skin hazard classification, according to the GHS systems and EU, are shown in Table 1. All acidic and basic materials, MTT (3-(4,5-dimethylthiazol-2-yl)-2,5-diphenyltetrazolium bromide), D-PBS, and sodium dodecyl sulfate (SDS), were purchased from Sigma–Aldrich (St. Louis, MO, USA) with the highest grade available. McIlvaine buffer consists of 0.1 M monohydrated citric acid and 0.2 M disodium hydrogen phosphate (Na_2_HPO_4_) prepared in different volume ratios to achieve the specific pH [14].

### 2.2. D-Reconstructed Human Epidermis Skin Model

The skin model used in the skin irritation tests, KeraSkin™ (Biosolution Co., Ltd., Seoul, Korea), is a commercially available reconstructed human epidermis model prepared from primary normal human keratinocytes [28]. The KeraSkin™ model was cultured to be a multilayered and fully differentiated human keratinocyte. DMEM-based maintenance medium was also provided along with KeraSkin™ by Biosolution Co. Upon delivery, KeraSkin™ was placed on a 6-well plate filled with 0.9 mL/well culture media, and pre-incubated for 22 ± 2 h at 37 °C and 5% CO_2_.

### 2.3. In Vitro Skin Irritation Test (SIT)

The protocol for the KeraSkin™ skin irritation test was adopted using the me-too test method for OECD TG 439 [29] and was used for the evaluation of skin irritation of various chemicals [30,31,32,33]. After the pre-incubation, the tissue was removed from the incubator (Thermo Scientific, Waltham, MA, USA), and the test materials were applied immediately. Then, 40 μL of acid or alkali diluted in deionized water (DW) at indicated concentrations was dispensed directly on the tissue surface, and sterile forceps were used to tilt the insert and gently spread the liquid. DW was used as the negative control and 5% SDS as the positive control. After the test chemicals were applied, the plates were incubated at 37 °C and 5% CO_2_. After 30 min, the tissues were rinsed with DPBS. Tissues were post-incubated for 42 ± 2 h and then the entire medium was removed. Tissues were blotted and transferred to a 24-well plate that contained MTT (0.4 mg/mL) and incubated for 3 h at 37 °C and 5% CO_2_ [34]. Next, tissues were transferred to a new 6-well plate, prefilled with 2 mL of isopropanol. Formazan extraction was performed at room temperature for 3 h, and 250 μL of formazan extract per tissue was transferred to a 96-well plate (SPL, Pocheon-si, Republic of Korea). Optical density (OD) was measured at 570 nm using isopropanol as a blank with a microplate spectrophotometer (BioTek Instruments, Inc., Winooski, VT, USA). No test chemicals appeared to react with MTT.

### 2.4. Histological Analysis

For the histological examination, after MTT extraction, KeraSkin™ samples were fixed in 10% neutral-buffered formalin. According to the previous study [35], preserved tissues from each group were paraffin-embedded, sectioned, stained with hematoxylin and eosin (H&E, Dako, Agilent, anta Clara, CA, USA), and then examined microscopically under an Olympus DP71 microscope (Center Valley, PA, USA). All images of tissues were obtained using the virtual slide system (Aperio Scanscope XT, Vista, CA, USA).

### 2.5. Visualization of Lipid Distribution in KeraSkin^TM^ with Nile Red Staining

Cryosections of KeraSkin^TM^ models which had not undergone the MTT assay were stained with Nile Red (Sigma–Aldrich) to visualize the distribution of lipids [36]. Before Nile Red was used, 15 μL stock solution (500 μg/mL Nile Red in acetone) was diluted in 1 mL of 75% glycerol (VWR International, Radnor, PA, USA), and a diluted staining solution was dropped on each biopsy specimen section. A glass coverslip was then placed over each slide, and the slides were left in the dark for 5 min before observation by Softmax 5.2 program and Axio Observer 7 microscope (Carl Zeiss, Oberkochen, Germany) equipped at the Ewha Drug Development Research Core Center.

### 2.6. Statistics

Data are expressed as the mean ± standard error of the mean (SEM) of three or more independent experiments. The statistical significance of differences between groups was assessed using a two-sided Student’s *t*-test. *p*-values < 0.05 were considered significant. 

## 3. Results

### 3.1. Skin Irritation of Acidic pH Determined in KeraSkin™

**Figure 1:**
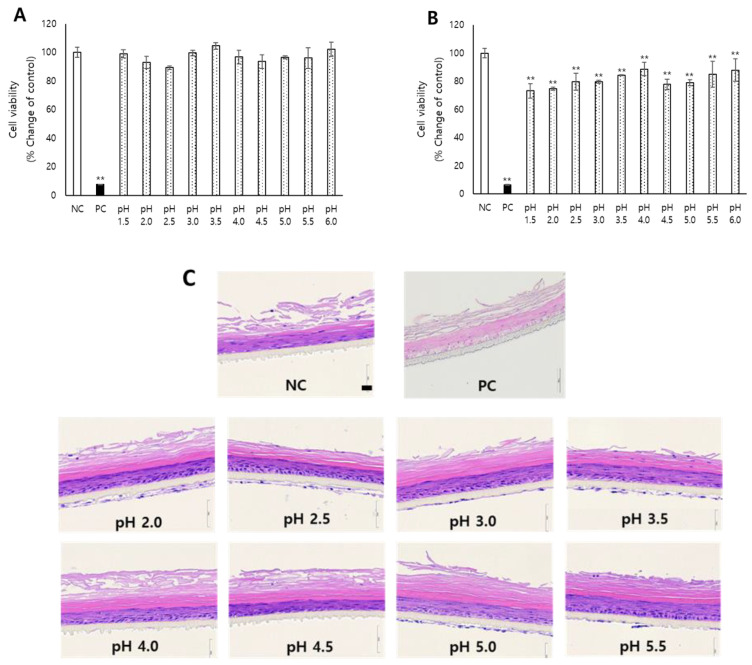
In this figure, 0.1 M McIlvaine buffers prepared with indicated pH ranges were treated with KeraSkin™ for (**A**) 30 and (**B**) 180 min, and cell viability was measured with an MTT assay. See Methods for detailed procedure. (**C**) KeraSkin™ treated with test chemicals was stained with H&E. Scale bar is 50 μm. NC; negative control (DW) with white bars, PC; positive control (5% SDS) with filled bars. McIlvaine buffers with dotted bars ** *p* < 0.01.

To examine the effect of acidic pH on skin irritation, the McIlvaine buffer, which had the widest pH range among the various biological buffers based on citric acids and phosphates, was employed. McIlvaine buffers have been widely used in biological tests to simulate a variety of conditions occurring in living organisms [37]. McIlvaine buffers prepared at 0.1 M in the range of pH 1.5–6.0 were applied for 30 min to KeraSkin^TM^. Surprisingly, there were no significant changes in cell viability at the tested pH (Figure 1A). After a longer exposure beyond the standard time (180 min exposure), the tissue viability was significantly reduced compared to the negative control, but the cell viability was preserved as higher than 50%, the cutoff for determining the test substance as a skin irritant. (Figure 1B). In line with this, the tissues treated for 180 min showed no significant difference in the histology from the negative control. 

### 3.2. Evaluation of Skin Irritation of Various Organic Acids 

**Figure 2:**
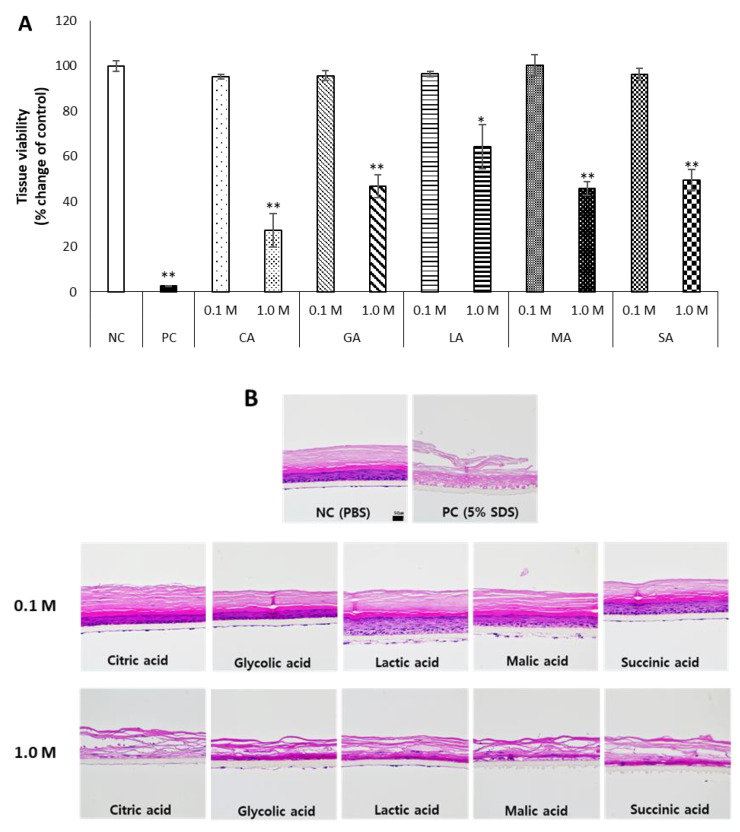
Various organic acids were prepared at a low concentration (0.1 M) and a high concentration (1.0 M) and exposed to KeraSkin™ for 30 min. Then, (**A**) cell viability was evaluated, and (**B**) skin tissues were stained with H&E. Scale bar is 50 μm. NC; negative control (DW) with white bars, PC; positive control (5% SDS) with filled bars. Tested samples with patterned bars, CA; citric acid, GA; glycolic acid, LA; lactic acid, MA; malic acid, SA; succinic acid. * *p* < 0.05, ** *p* < 0.01.

The acid components of McIlvaine buffer are citric acid and phosphoric acid. To examine whether the anionic part may affect the skin irritation of acidic substances, various organic acids commonly used in the cleaners [37] were tested at low (0.1 M) and high concentrations (1.0 M). This concentration range is close to the maximum concentrations of acids used in cleaners [38]. The organic acids selected were citric acid, glycolic acid, lactic acid, malic acid, and succinic acid. Their pH range was 1.89–2.60 at 0.1 M and 1.13–1.82 at 1 M (Table 1). When acids were tested for skin irritation using KeraSkin™, there was no significant decrease in cell viability at 0.1 M. However, at 1 M, it was confirmed that the organic acids significantly reduced the viability to below 50%, except for lactic acid (Figure 2A).

KeraSkin™ tissues remaining after the MTT assay were processed and stained with H&E. When treated with low concentrations of organic acids, the stratified structures of the stratum corneum, stratum granulosum, stratum spinosum, and stratum basale were relatively well preserved, similarly to the negative control. However, when high concentrations of organic acids were treated, the stratified structure of the epidermis was mostly destroyed, which was more severe than the pattern shown in the tissue viability. Interestingly, compared to other skin layers, the stratum corneum maintained its shape relatively well (Figure 2B). 

### 3.3. Evaluation of Skin Irritation of Various Inorganic Acids 

**Figure 3:**
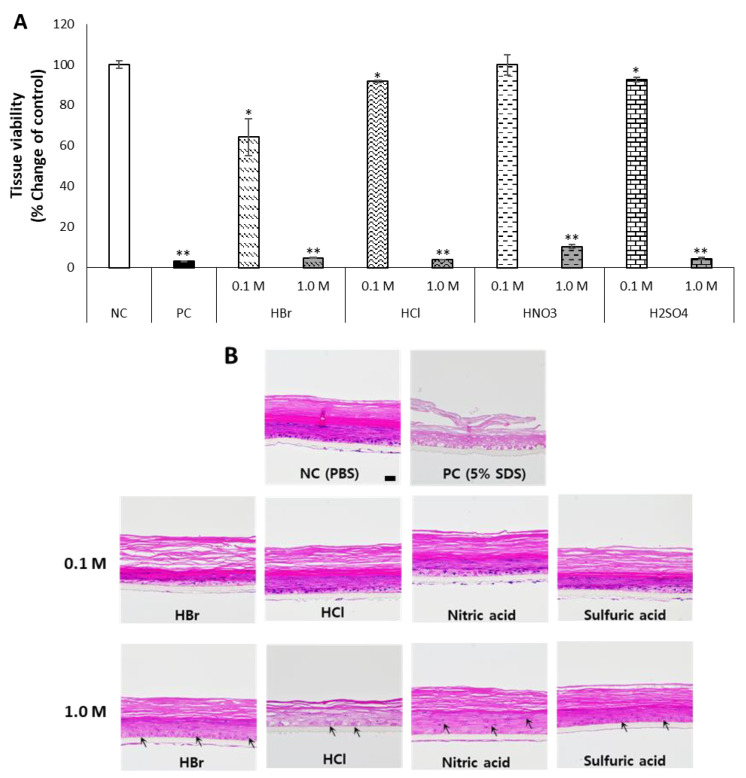
Inorganic acids were prepared at a low concentration (0.1 M) and a high concentration (1.0 M) in DW and exposed to KeraSkin™ for 30 min. Then, (**A**) cell viability was evaluated, and (**B**) skin tissues were stained with H&E. Scale bar is 50 μm. NC; negative control (DW) with white bars, PC; positive control (5% SDS) with filled bars. Tested samples with patterned bars, HBr; hydrogen bromide, HCl; hydrogen chloride, HNO_3_; nitric acid, H_2_SO_4_; sulfuric acid. Arrows show vacuolization and pyknosis. * *p* < 0.05, ** *p* < 0.01.

In order to determine the effect of inorganic acids, inorganic acids composed of a single component at low (0.1 M) and high concentrations (1.0 M) were evaluated for skin irritation (Figure 3).

The inorganic acids selected were hydrogen chloride (HCl), hydrogen bromide (HBr), nitric acid (HNO_3_), and sulfuric acid (H_2_SO_4_). Their pH range was 0.47–0.69 at 0.1 M and −0.66–−0.29 at 1 M. The pH of inorganic acids was mostly lower than that of organic acids (Table 1). When inorganic acids were treated at 0.1 M, while the cell viability was maintained above the 50% cutoff, the skin tissue showed more damaged patterns compared to those treated with the organic acids. At 1 M, the irritation was pronounced, and all the inorganic acids tested were classified as skin irritants with respect to cell viability (Figure 3A). Additionally, in the histological analysis, the thickness of the skin layers except for the stratum corneum was slightly thinned at 0.1 M. The tissue treated with 1 M of inorganic acids showed vacuolization and pyknosis from necrosis in most of the skin layers. The cell layers, especially stratum granulosum, stratum spinosum, and stratum basale, were much thinned, and it was almost impossible to distinguish each cell layer. Interestingly, the thickness and shape of the stratum corneum were relatively well preserved (Figure 3B).

### 3.4. Evaluation of Skin Irritation of Various Organic and Inorganic Alkalis

In order to compare the effects of alkaline pH with acids, organic and inorganic alkalis used in cleaning agents were investigated. Two of the most widely used organic and inorganic alkaline substances were selected and their toxicity was evaluated, and the materials used in the experiment are shown in Table 1. 

**Figure 4:**
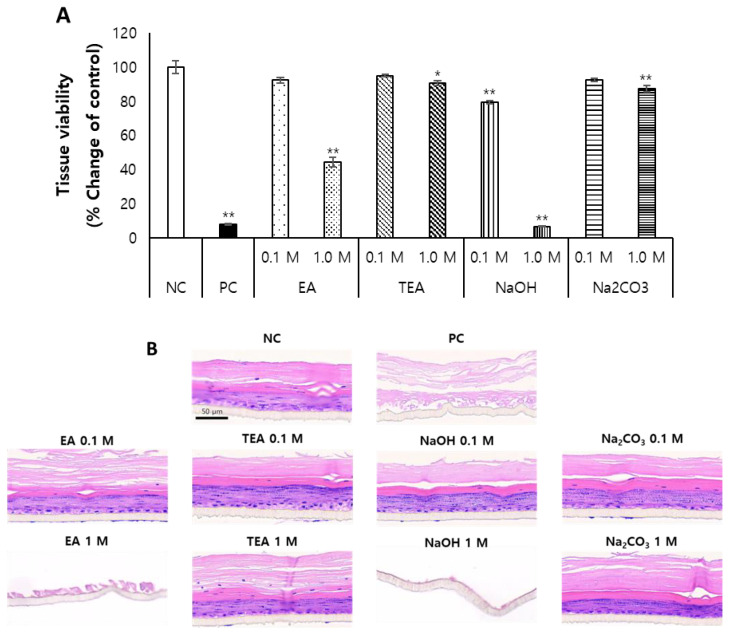
Various organic and inorganic alkalis were prepared at a low concentration (0.1 M) and a high concentration (1.0 M) and exposed to KeraSkin™ for 30 min. Then, (**A**) cell viability was evaluated, and (**B**) skin tissues were stained with H&E. Scale bar is 50 μm. NC; negative control (DW) with white bars, PC; positive control (5% SDS) with filled bars. Tested samples with patterned bars, EA; ethanolamine, TEA; triethanolamine, NaOH; sodium hydroxide, Na_2_CO_3_; sodium carbonate. * *p* < 0.05, ** *p* < 0.01.

When the alkaline solutions were evaluated for skin irritation with KeraSkin^TM^, it was revealed that both the pH (>12) and concentration (>0.1 M) are key to the irritancy of alkaline substances, as demonstrated by the cases of ethanolamine (EA) and sodium hydroxide (NaOH). As shown in Figure 4A, EA was not an irritant at 0.1 M (pH 11.4), but at 1 M (pH 12.0), it was a skin irritant. In the case of NaOH, it was not irritant at 0.1 M even though the pH was 13.07. NaOH showed skin irritancy only at 1.0 M (pH 13.68).

KeraSkin™ tissues remaining after the MTT assay were processed and stained with H&E (Figure 4B) which supports the cell viability results.

### 3.5. Effects of Acid and Alkali Substances on the Lipid Composition of KeraSkin^TM^

**Figure 5:**
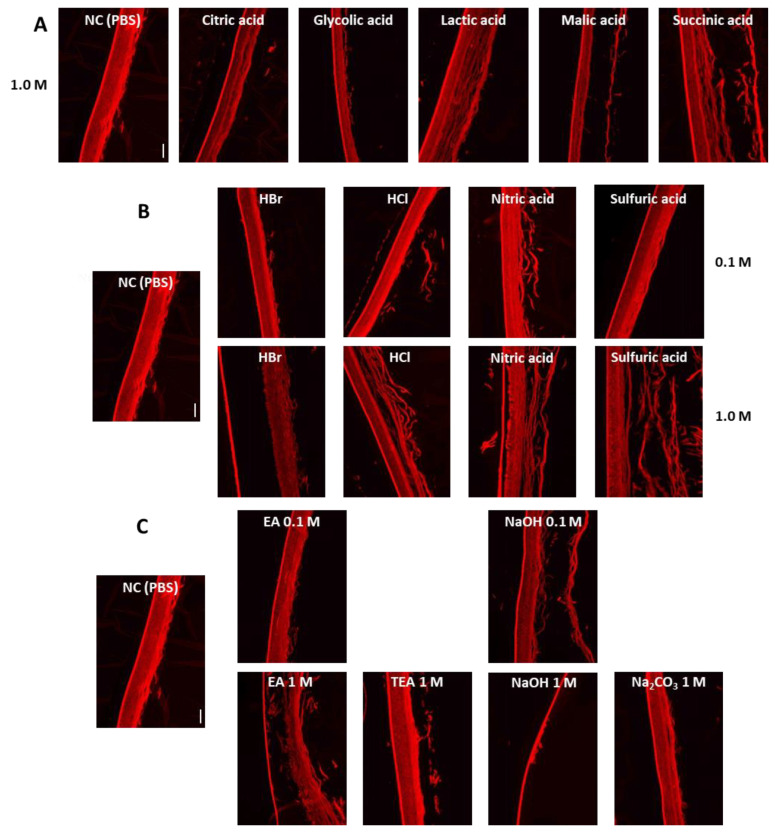
Distribution of lipids in the treated tissues was examined by Nile Red staining. Representative fluorescent microscope image with x magnification. Scale bar is 100 μm. (**A**) Organic acids, (**B**) inorganic acids, and (**C**) organic and inorganic alkalis were treated.

To investigate the effects of acid and alkali substances on the lipid composition of KeraSkin^TM^, treated tissues were stained with a lipid-specific fluorescent dye, Nile Red. As shown in Figure 5, organic acids at irritant concentrations depleted the epidermal lipids, as shown by the paler Nile Red staining, but the overall lipid structure was preserved relatively intact. However, inorganic acids at irritant concentrations not only depleted epidermal lipids but destroyed the overall lipid structures. In the case of alkali, the tissues were more severely damaged than those treated with acids with badly destroyed lipid structures even at non-irritant concentrations.

## 4. Discussion

Here we investigated whether acidic pH plays a direct role in determining the skin irritation of acidic agents. Our results demonstrated that acidic pH alone cannot be the sole factor to induce skin irritation, but the concentrations and types of anions are also important in determining the skin irritancy of acids. 

Here we could observe that the tissues treated with acids had a well-preserved stratum corneum (SC), although the underlying epidermal layers were damaged after the treatment with high concentrations of acids. This feature is completely different from the cases of other types of skin irritants such as surfactants or alkaline agents, wherein the SC is the primary target of the skin epidermis. Ananthapadmanabhan et al. also showed that acidic pH 4.0 induced the shrinking of SC but in a well-organized fashion [10]. The SC is a barrier that prevents the entry of environmental pollutants and pathogens into the body. Furthermore, it prevents excessive water loss from deeper skin layers into the environment [39]. Therefore, the loss of SC integrity is considered to be a critical and primary step for the manifestation of skin irritancy [40]. The formation of the SC requires pH-dependent enzymes such as acid hydrolase, β-glucocerebrosidase, acid sphingomyelinase, acid lipases, phosphatases, and phospholipases [6]. These enzymes need a milieu of acidic pH to properly function. Indeed, the SC is maintained at a slightly acidic pH ranging from 5.4–5.9 supporting the suggestion that the SC may be more resistant to acidic substances.

It was reported that alkaline pH increases SC swelling and impairs the fluidity of SC lipids [41]. We could also observe that the tissues treated with alkali agents showed badly damaged SC lipids, the extent of which was more severe than those treated with acids, supporting that high alkaline pH can be more detrimental to the organization of SC lipids as well as SC protein structure. In addition, it is generally accepted that skin irritation by surfactants is caused by their penetration into living epidermis through the SC and the subsequent disruption of epidermal cells [42]. For this reason, SC is seriously damaged by surfactants as an early event of skin irritation, as can be seen with the images of tissues treated with the positive control, SDS. Conversely, acidic cleaners result in more minimal disruption of the skin’s barrier [43]. 

In this study, the skin irritation was remarkable only at high concentrations (>0.1 M) of organic and inorganic acids. When high concentrations of organic acids were treated on skin tissues, the stratified structure of the epidermis was severely damaged. Considering that the SC was relatively intact, we consider that acids permeated through the SC without damaging it, but affected the inner layer of viable keratinocyte layers. Indeed, fatty acids are known to enhance transdermal delivery by perturbing the SC lipid structure [44], which may explain our results.

A physicochemical approach to predict the skin irritancy of a substance was tried based on the determination of the pH of the acidic and alkaline substances [45]. Worth and Balls studied prediction models based on pH values to predict the potential of chemicals to cause skin corrosion and irritation [46]. The prediction models have a clear mechanistic interpretation: Chemicals that form acidic or basic solutions are likely to be corrosive or irritating [45]. This supports the need to consider the acidic and basic properties of chemicals during the assessment of skin irritancy [47]. However, these models used highly concentrated substances (over 10 M) for the construction of the base data and a number of chemicals with intermediate pH values are also determined to be corrosives or skin irritants [45], which indicates the existence of mechanisms other than pH. Further supporting the importance of these types of acids, ECHA database records based on in vivo data suggest that many of acids were indeed not irritating or mildly irritating, which is in line with our data.

## 5. Conclusions

Collectively, we demonstrated that the skin irritancy of acidic substances is not simply determined by acidity only, but by a number of other factors such as the type of anions, concentration, and duration of skin exposure. We believe that further studies are necessary to fully characterize the factors determining the skin irritation of acidic substances to develop safer cleaners. 

## Figures and Tables

**Table 1:** Information on acid and alkali agents tested.

Chemical	IUPAC Name	CAS No.	Molecular Weight	Molecular Formula	Purity	Water Solubility ^b^	pH (Skin Irritation ^a^)		ECHA^b^	
							0.1 M	1.0 M	Skin hazard	Source
*Organic acid*										
Citric acid	2-Hydroxypropane-1,2,3-tricarboxylic acid	77-92-9	192.12	C_6_H8O_7_	≥97%	540 g/L	1.89 (NI)	1.13 (I)	Mildly irritating (0.5 g powder)	[15]
Glycolic acid	2-Hydroxyacetic acid	79-14-1	76.05	C_2_H_4_O_3_	99%	> 300 g/L	2.41 (NI)	1.61 (I)	Skin Corr. 1B (99% liquid)	[16]
Lactic acid	2-Hydroxypropanoic acid	50-21-5	90.08	C_3_H_6_O_3_	88–92%	861 g/L	2.43 (NI)	1.60 (NI)	Mildly irritating (88% aqueous solution)	[17]
Malic acid	2-Hydroxybutanedioic acid	6915-15-7	134.09	C_4_H_6_O_5_	≥99%	647 g/L	2.25 (NI)	1.43 (I)	Mildly irritating (0.5 g wetted with paraffin oil)	[18]
Succinic acid	Butanedioic acid	110-15-6	118.09	C_4_H_6_O_4_	≥99%	83 g/L	2.60 (NI)	1.82 (I)	Not irritating (0.5 g moistened with water)	[19]
*Inorganic acid*										
Hydrogen bromide	Bromane	10035-10-6	80.91	HBr	30–32% in acetic acid	665 g/L	0.55 (NI)	−0.66 (I)	Skin Corr. 1A	[20]
Hydrogen chloride	Chlorane	7647-01-0	36.46	HCl	37% aq. solution	500 g/L	0.69 (NI)	−0.29 (I)	Skin Corr. 1A (25~30%)	[21]
Nitric acid	Nitric acid	7697-37-2	63.01	HNO_3_	70% aq. solution	>500 g/L	0.58 (NI)	−0.39 (I)	Skin Corr. 1A (≥28%)	[22]
Sulfuric acid	Sulfuric acid	7664-93-9	98.08	H_2_SO_4_	95–98%	1000 g/L	0.47 (NI)	−0.55 (I)	Skin Corr. 1A (Predicted)	[23]
*Organic alkali*										
Ethanolamine	2-Aminoethanol	141-43-5	61.08	C_2_H_7_NO	≥99%	1000 g/L	11.40 (NI)	12.00 (I)	Skin Corr. 1B (Neat liquid, 20% solution)	[24]
Triethanolamine	2-[Bis(2-hydroxyethyl)amino]ethanol	102-71-6	149.19	C_6_H_15_NO_3_	≥99%	1000 g/L	10.36 (NI)	10.90 (NI)	Not irritating (0.5 mL neat)	[25]
*Inorganic alkali*										
Sodium hydroxide	Sodium hydroxide	1310-73-2	39.997	HNaO	≥97%	1000 g/L	13.07 (NI)	13.68 (I)	Skin Corr. 1A (>2%)	[26]
Sodium carbonate	Disodium carbonate	497-19-8	105.988	Na_2_CO_3_	≥99.5%	212.5 g/L	11.49 (NI)	11.60 (NI)	Not irritating (0.5 g)	[27]

^a^ NI; Non-irritant, I; irritant. ^b^ ECHA database accessed through eChemportal on 19 September 2022 [15].

## Data Availability

The data presented in this study are available on request from the corresponding author.
